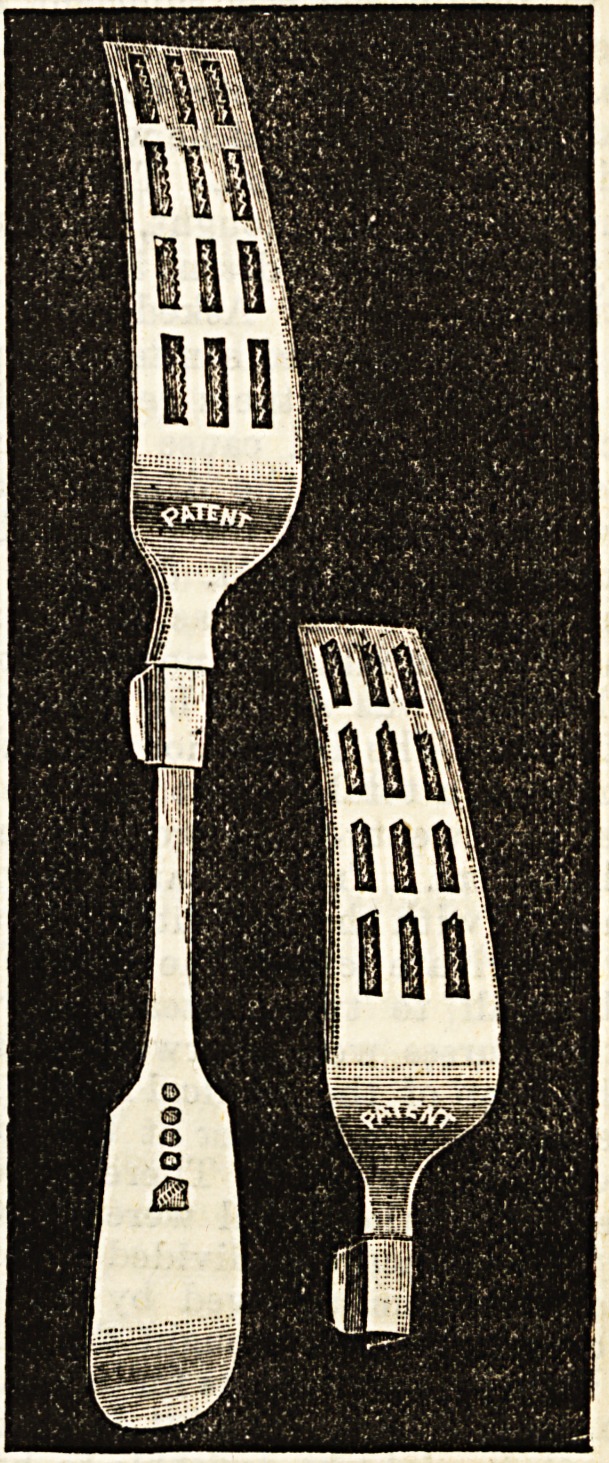# New Drugs, Appliances, and Things Medical

**Published:** 1891-03-07

**Authors:** 


### NEW DRUGS, APPLIANCES, AND THINGS MEDICAL.

[All preparations, appliances, novelties, etc., of which a notice is desired, should be sent for The Editor, to care of The Manager, 140, Strand, London, W.C.]

### THE MASTICATOR.

This is an instrument to enable the dyspeptic to properly prepare his meat before putting it into his mouth. It consists of a shield or fork-like case perforated with a series of slots on the underside of which is an attachment fitted with twelve fine teeth-like cutters, arranged in four groups of three, which project through the slots or openings of the case into which they are set. Both parts are readily and securely fitted over the fork, and are easily removed for cleaning. The masticator when in use should be held in the right hand, the teeth or cutting edges being drawn through the meat or other food with a moderate degree of pressure. The use of an ordinary fork in the other hand is necessary to hold the meat firmly and to convey it to the mouth when properly prepared by the masticator. We have made a trial of the specimen forwarded to us by the Crysolite Gold Company, Chancery Lane, and find that it answers its purpose extremely well. We find the points in its favour are: (1) Efficiency. It divides meat into a fine pulp. (2) Ease of use. (3) Lowness of price and also the cheapness of supplying new cutting attachments, as six new ones only cost a shilling.

### HORLICK'S MALTED MILK.

We have received from 39, Snow Hill samples of the above. It consists of a cream-coloured granulated powder with the odour of well-dried malt. On agitating with warm water it dissolves, forming a milky fluid. Our analysis shows this to be a valuable preparation, well made, and likely to prove useful in the many cases where liquid food is necessary. It is by no means unpleasant or "sickly" to the taste, and being very nutritious will do well as an occasional variant of tea, coffee, or cocoa. It contains the constituents of milk, and has the digestive powers of malt, therefore it is well adapted for cases where the assimilative powers are weak

## Figures and Tables

**Figure f1:**